# Impact of Educational and Training Programs on Knowledge of Healthcare Students Regarding Nosocomial Infections, Standard Precautions and Hand Hygiene: A Study at Tertiary Care Hospital

**DOI:** 10.5005/jp-journals-10071-23166

**Published:** 2019-05

**Authors:** Mohit Goyal, Dhruva Chaudhry

**Affiliations:** 1-2 Department of Pulmonary and Critical Care Medicine, Post Graduate Institute of Medical Sciences, Rohtak, Haryana, India

**Keywords:** Hand hygiene, Knowledge, Nosocomial infections, Standard precautions

## Abstract

**Background and Objectives:**

Nosocomial infections are significant public health problems in developed as well as developing countries. To tackle this problem, it is vital to sensitize healthcare students (HCSs) at early period of their clinical practise. Thus, this study was conducted to access the existing knowledge among HCSs and determine the impact of educational and training programs regarding nosocomial infections, standard precautions, and hand hygiene.

**Methods:**

This was a cross-sectional cum interventional, questionnaire based, single center study. Total 728 MBBS, BDS and BSC nursing students were targeted for workshop on nosocomial infections, standard precautions, and hand hygiene based on the Centers for Disease Control and prevention (CDC) and World Health Organization (WHO) guidelines. Infection control standardized questionnaire (ICSQ) was administered as a pretest and posttest. Results were analyzed by SPSS software.

**Results:**

A paired-samples t-test was conducted to access the impact of educational and training programs on knowledge of HCS. There was a significant difference in the scores of pretests (M = 37.30, SD = 4.81) and posttests (M = 42.03, SD = 4.55); t (727) = 22.162, *p* ≤0.005 and also statistically significant difference in scores of all 3 domains- 1. Nosocomial infections: Pretest (M = 6.61, SD = 1.57) and Posttest (M = 7.98, SD = 4.65); t (727) = 20.589, *p* ≤ 0.005; 2. Standard Precautions: Pretest (M = 20.81, SD = 3.06) and Posttest (M = 41.88, SD = 4.30); t (727) = 4.584, *p* ≤ 0.005; 3. Hand Hygiene: Pretest (M = 9.88, SD = 2.68) and Posttest (M = 12.54, SD = 2.92); t (727) = 19.527, *p* ≤ 0.005. The results suggest that educational and training programs have positive impact on knowledge of HCS.

**Conclusion:**

This study highlighted the need for regular educational and training programs in primary training time for retention of knowledge regarding nosocomial infections and reinforcement of the principals of standard precautions and hand hygiene.

**How to cite this article:**

Goyal M, Chaudhry D. Impact of Educational and Training Programs on Knowledge of Healthcare Students Regarding Nosocomial Infections, Standard Precautions and Hand Hygiene: A Study at Tertiary Care Hospital. Indian J Crit Care Med 2019;23(2):227–231.

## BACKGROUND

Nosocomial infections are localized or systemic infections due to infectious agents or their toxins which are not present or incubating at the time of admission of patient in heath care center.^1–3^ These infections are significant public health problems in developed as well as developing countries. More than 1.4 million people all over world are suffering from infections acquired during hospital stay.^2^ Among patients admitted to health centers^4–6^ in India, studies reveal that 10–20% of the patients admitted had acquired nosocomial infections. Most healthcare centers in developing countries lack effective infection control programs, posing additional risk.^3^ The presence of nosocomial infection further worsens the patient condition thus increasing the time of hospitalization, patient agony, and healthcare costs.^7^

Standard precautions including the infection control measures, if used strictly by the healthcare workers, can significantly contribute to reduction in spread of pathogens.^8^ The most important and effective component of this protocol is hand hygiene, which suggest basic practices of hand washing, hand sanitization, and use of gloves.^9^ These basic measures have always been shown to prevent infection spread regardless of the patient symptoms. Few studies conducted in France^10^, Ghana^11^, Ethiopia^12^, and India^13^ show that there is need for more emphasis on education about sources of nosocomial infection. They also suggest that besides knowing basic guidelines, there is non-adherence to standard precautions and hand washing practices among healthcare workers. They recommend educating HCS about these concepts and formulation of guidelines for every hospital.

Therefore, it is vital for HCS, patient attendants, and patient themselves to be sensitized to principles of nosocomial infection. More importantly these skills would be useful for HCS as they begin clinical practice. Additionally, early interventions improving the awareness at all these patient contact levels regarding spread and control techniques are important to reduce the burden of such infections, ensuring better quality healthcare. Therefore, we planned a study to assess existing knowledge of healthcare students regarding nosocomial infection and effects of interventions to existing knowledge base.

### METHODS

This was a cross-sectional cum interventional, questionnaire based, single center study carried out at a tertiary care hospital of India. Ethical clearance was obtained prior to the start of study. Total 728 MBBS, BDS and BSc nursing students attending clinical postings participated in this study with characteristics mentioned above. Students were informed about the study procedure, privacy, confidentiality, and voluntary participation in the study following which a written informed consent was obtained.

Characteristics of study population are presented in Table 1.

**Fig. 1:**
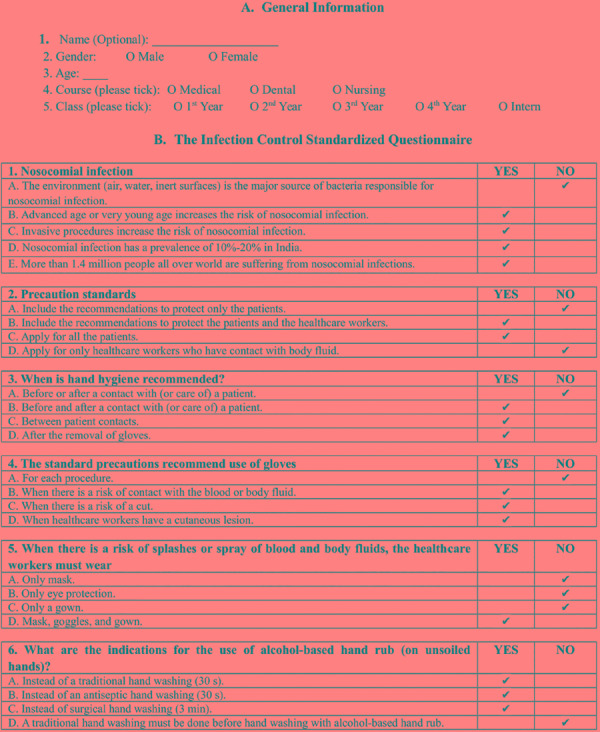
Research tool: Infection Control Standardized Questionnaire (ICSQ)

*Research tool:* (Fig. 1) Infection Control Standardized Questionnaire (ICSQ)^10^ with Cronbach α test (internal consistency coefficient) value of 0.61 was used.

1st part collected basic demographic information of each participant.

2nd part consisted of some questions collecting information in the following three main domains, with 25 close-ended questions:

- Nosocomial infection (5)
- Standard precautions (12)
- Hand hygiene (8)

Response to each item was coded and scored as a

- Correct answer = 2
- Don't know = 1
- Incorrect answer = 0
  The maximum score for this questionnaire was 50.

**Table 1:** Characteristics of study population

				*Age*	
*Course*	*Year*	*Gender*		*Mean*	*Standard deviation*
		*Male*	*Female*		
MBBS	2nd	92	69	20.11	1.30
	3rd	100	84	21.24	1.07
	4th	55	21	22.74	1.20
	Subtotal	247	174	21.08	1.51
BDS	1st	5	17	19.00	.76
	2nd	3	17	21.20	.77
	3rd	10	11	21.71	1.01
	4th	8	19	22.33	1.00
	Subtotal	26	64	21.12	1.56
BSc Nursing	1st	0	39	18.69	.86
	2nd	0	58	19.64	.81
	3rd	0	60	20.43	.81
	4th	0	60	21.32	.72
	Subtotal	0	217	20.15	1.22

#### Procedure

All the participants of the study were scheduled for a brief lecture cum workshop in small batches. This included a didactic session of 30 minutes covering the key concepts of nosocomial infections, universal standard precautions, and hand washing based on CDC^14^ and WHO^15^ guidelines. This session was followed by practical demonstration and hands-on training of taught concepts, emphasizing hand hygiene procedures. ICSQ was administered as a pretest and posttest to assess the knowledge of the participants.

#### Analysis

The data collected was entered into a Microsoft Excel spread sheet and statistical analysis was performed using Statistical Package for Social Sciences (SPSS) version 23.0. Intergroup comparison of continuous and categorical variables was performed using paired samples t-test. Assessment of differential levels of knowledge in each domain (nosocomial infections, standard precautions, and hand hygiene) with respect to course of participants was done. For all test, significance level was kept at *p* <0.05.

### RESULTS

**Fig. 2:**
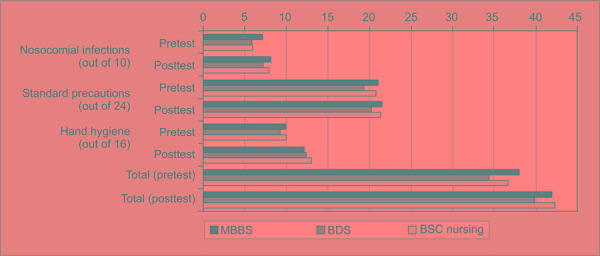
Comparison of mean scores of each domain

Evaluation of pretest and posttest of each HCS was done and mean ± SD of all 3 domains was calculated for MBBS, BDS and BSC nursing students as shown in Table 2 and Figure 2.

A paired-samples t-test (Table 3) was conducted to access the impact of educational and training programs on knowledge of HCS. There was a significant difference in the scores of pretests (M = 37.30, SD = 4.81) and posttests (M = 42.03, SD = 4.55); t (727) = 22.162, *p* ≤0.005. There was statistically significant difference in scores of each domain also:

- *Nosocomial infections:* Pretest (M = 6.61, SD = 1.57) and Posttest (M = 7.98, SD = 4.65); t (727) = 20.589, *p* ≤0.005
- *Standard Precautions:* Pretest (M = 20.81, SD = 3.06) and Posttest (M = 41.88, SD = 4.30); t (727) = 4.584, *p* ≤0.005
- *Hand Hygiene:* Pretest (M = 9.88, SD = 2.68) and Posttest (M = 12.54, SD = 2.92); t (727) = 19.527, *p* ≤0.005

These results suggest that educational and training programs have positive impact on knowledge of HCS regarding all three domains—nosocomial infections, standard precautions, and hand hygiene.

#### DISCUSSION

This study highlighted the important public health issue of nosocomial infections and the importance of standard precautions and hand hygiene in clinical training and hospital infection control programs. All the healthcare students attending clinical postings at a tertiary healthcare center were targeted. Their existing knowledge and awareness were accessed by administering the ICSQ as pretest. MBBS students had the highest mean score (out of 50) of 38.10, BSc Nursing students had mean score of 36.82, BDS students had mean score of 34.61. An intervention of organizing an educational and training program under CDC^14^ and WHO^15^ guidelines was done. The session was not only aimed at giving theoretical knowledge, rather a two-way participation was aimed by giving practical demonstration and hands-on training of taught concepts and emphasizing hand hygiene procedures. After the session, ICSQ was again administered as posttest to access the effectiveness of the training program. In posttest, BSC Nursing students came up with highest mean score of 42.40 followed by MBBS students (42.03) and BDS students (39.96). The impact of training session was significant and maximum on BDS students with 15.46% increase in knowledge status followed by BSc Nursing students (15.12% increase) and MBBS students (10.31% increase). These results highlight that there is a positive impact on HCS knowledge and compliance by timely training them with hands-on practice despite of only giving one-way lectures. Suchitra and Lakshmi^13^ in their study advised a yearly educational program for retention of knowledge, attitude, and practices among various categories of healthcare workers. The same implies for the healthcare students for the reinforcement of the principals of standard precautions and hand hygiene. For this, the primary training is best time frame as they are like unbaked pitcher and can be molded into perfect shape easily.

**Table 2:** Results of pretest and posttest in all 3 domains

	*Domains*	*Test*	*MBBS*	*BDS*	*BSc nursing*	*All HCSs*
Nosocomial Infections (out of 10)	Pretest	Mean	7.08	5.93	5.98	6.61
		SD	1.51	1.51	1.38	1.57
	Posttest	Mean	8.15	7.22	7.96	7.98
		SD	1.23	1.36	1.37	1.32
Standard precautions (out of 24)	Pretest	Mean	21.10	19.34	20.85	20.81
		SD	3.21	3.10	2.57	3.06
	Posttest	Mean	21.62	20.19	21.34	21.36
		SD	2.45	2.72	1.89	2.37
Hand hygiene (out of 16)	Pretest	Mean	9.93	9.33	10.00	9.88
		SD	2.76	2.48	2.58	2.68
	Posttest	Mean	12.25	12.54	13.10	12.54
		SD	3.12	2.81	2.45	2.92
Pretest (out of 50)	Mean	38.11	34.61	36.83	37.30	
	SD	4.81	3.84	4.15	4.65	
Posttest (out of 50)	Mean	42.03	39.96	42.40	41.88	
	SD	4.55	4.47	3.47	4.30	

**Table 3:** Paired samples t-test

	Paired differences (Posttest - Pretest)							
				95% confidence interval of the difference				
*Domains*	*Mean*	*Std. deviation*	*Std. error mean*	*Lower*	*Upper*	*t*	*Degree of freedom*	*p value*
Nosocomial infections	1.369	1.794	.066	1.238	1.500	20.589	727	≤.005
Standard precautions	.550	3.242	.120	.314	.786	4.584	727	≤.005
Hand hygiene	2.663	3.680	.136	2.395	2.931	19.527	727	≤.005
Total	4.586	5.583	.206	4.180	4.992	22.162	727	≤.005

### CONCLUSION

There is a need for regular educational and training programs for retention of knowledge regarding nosocomial infections and reinforcement of the principals of standard precautions and hand hygiene. This is expected to significantly reduce morbidity and mortality rate due to nosocomial infections as these students are the future of healthcare manpower. Due to sensitization and reinforcement, compliance to follow standard precautions will increase. For this, primary training is best time frame to inculcate the good habits of hand hygiene and to motivate them for practicing standard precautions religiously.
